# Human arylamine *N*-acetyltransferase 2 genotype-dependent protein expression in cryopreserved human hepatocytes

**DOI:** 10.1038/s41598-020-64508-0

**Published:** 2020-05-05

**Authors:** Raúl A. Salazar-González, Mark A. Doll, David W. Hein

**Affiliations:** Department of Pharmacology & Toxicology and James Graham Brown Cancer Center, University of Louisville School of Medicine, Louisville, Kentucky 40202 USA

**Keywords:** Biological techniques, Genetics

## Abstract

Human *N-*acetyltransferases (NAT; EC 2.3.1.5) catalyze the *N*-acetylation of arylamine and hydrazine drugs and the *O*-acetylation of *N*-hydroxylated metabolites of aromatic and heterocyclic amines. Two different isoforms of this protein, *N*-acetyltransferase 1 (NAT1) and *N*-acetyltransferase 2 (NAT2), are expressed in human hepatocytes. Both are encoded by a single 870-bp open reading frame that exhibits genetic polymorphisms in human populations. NAT1 and NAT2 share more than 85% gene and protein sequence, making it challenging to produce antibodies with high specificity for NAT1 or NAT2. In the present study, we compared methods for the quantification of immunoreactive NAT1 and NAT2 with seven different antibodies and investigated the relationship of NAT2 genotype to NAT2 mRNA and protein expression in cryopreserved human hepatocytes. Sulfamethazine (NAT2-selective substrate) and NAT2 protein expression differed significantly with NAT2 acetylator genotype (p < 0.0001). NAT2 protein expression and sulfamethazine NAT2 catalytic activity correlated highly across the cryopreserved human hepatocytes of rapid, intermediate, and slow acetylator NAT2 genotypes. In conclusion, our data describe a specific analytical method for the quantification of NAT1 and NAT2 protein expression. We showed that the NAT2 activity in human hepatocytes is directly correlated to expression levels of NAT2 protein but not mRNA.

## Introduction

Arylamine *N*-acetyltransferases (NAT; EC 2.3.1.5) are xenobiotic metabolizing enzymes with an essential role in the metabolism of many drugs and carcinogens^1^. They are encoded by two individual genes located on Chromosome 8.22. *N*-acetyltransferase 1 (NAT1) and *N*-acetyltransferase 2 (NAT2) share 87% and 81% of sequence homology at the mRNA and protein level, respectively. Both proteins have 290 amino acids with a molecular mass of 33.9 and 33.6 kDa, respectively. Despite their high similarity, NAT1 and NAT2 exhibit different substrate specificity and tissue distribution. It has been demonstrated in multiple studies a consistent expression of NAT1 transcript in all tissue types, major organs and blood cells^2^. On the other hand, NAT2 transcript expression shows a characteristic pattern, being highest in the liver, followed by the small intestine and colon^3,4^. Single Nucleotide Polymorphisms (SNPs) in NAT2 result in rapid, intermediate and slow *N*-acetylation phenotypes^5^.

The basis of interindividual variability in the NAT2 acetylation polymorphism were first described in tuberculosis patients being treated with isoniazid^6^. This finding gained relevance with the discovery that many hydrazines and arylamine drugs and carcinogens were affected by the same genetic polymorphism, thus having an effect on the toxicity, therapeutic efficiency or carcinogenesis risk of such compounds^7,8^. Arylamine carcinogens require metabolic activation in order to initiate carcinogenesis. This process often is initiated by *N*-oxidation to form *N*-hydroxy aromatic or heterocyclic intermediates that are then subject to an *O*-acetylation reaction to form acetoxy intermediates capable of the spontaneous formation of DNA adducts^9^. NAT2 catalyzes both *N-*acetylation and *O-*acetylation reactions; modifying carcinogenesis risk with respect to NAT2 genotype, as demonstrated for carcinogens like 4-aminobiphenyl (ABP) in breast and urinary bladder cancer risk^10–13^.

Several reports have demonstrated the NAT2 genotype-dependent differences in *N-*acetylation capacity for different substrates. However, the role of NAT2 genotype on NAT2 protein expression has not been reported in human tissues. Although a previous report in human liver biopsies reported a decrease in the amount of immunodetectable NAT2 protein in subjects with a slow acetylation phenotype^14^, the present work focused on the quantification of NAT2 protein expression in cryoplateable human hepatocytes and its relationship to NAT2 genotype and NAT2 catalytic activity.

## Results

### Expression of NAT1 and NAT2 mRNA in UV5/NAT1 and UV5/NAT2 cells

**Figure 1:**
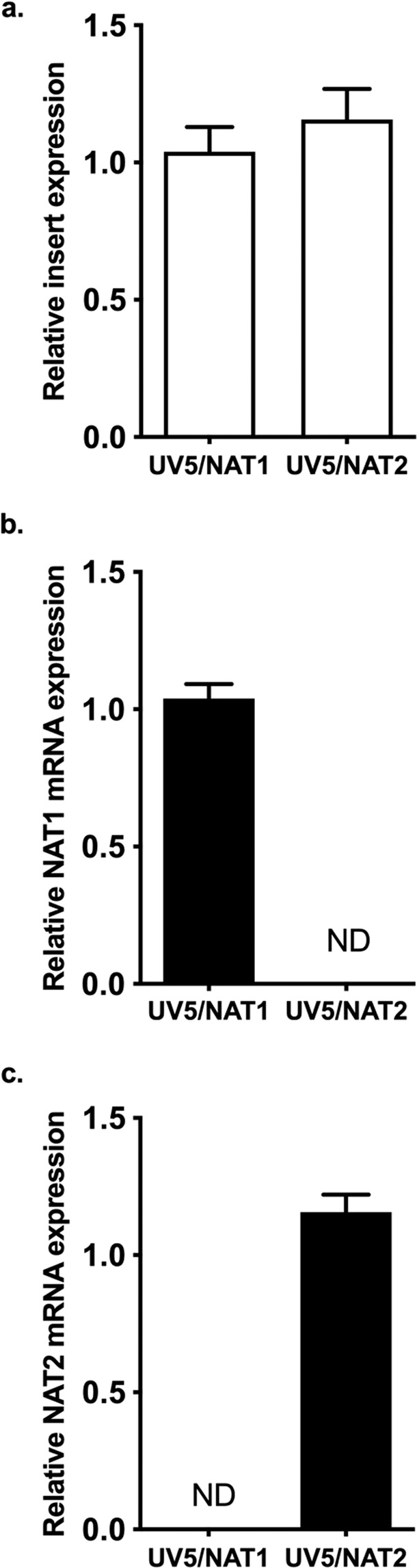
NAT1 and NAT2 mRNA expression in the UV5/NAT1 and UV5/NAT2 cells. The relative mRNA expression of NAT1 and NAT2 was quantified as described in the methods section. Each bar illustrates the mean ± SEM of relative mRNA expression from three individual determinations. (**a**) Relative expression of both NAT1 and NAT2 inserts in the UV5 cells. No difference in the expression was observed (p > 0.05). (**b**) Relative expression of NAT1 mRNA in both UV5 cell lines; NAT1 transcript was not detected in the UV5/NAT2 cells. (**c**) Relative expression of NAT2 mRNA in both UV5 cell lines; NAT2 transcript was not detected in the UV5/NAT1 cells.

In order to assess the expression of the inserted gene, we quantified the mRNA expression of both genes, in both UV5/CHO cell lines. No differences were shown in the relative expression of NAT1 insert or NAT2 insert in the respective cells (Fig. 1a), thus allowing us a direct comparison of the expression of both proteins. In order to assess the cross-mRNA expression of NAT2 in UV5/NAT1 cells, and NAT1 expression in UV5/NAT2 cells, respectively, the expression of both proteins was evaluated in both cell lines. No detectable expression of NAT2 was found in UV5/NAT1 cells (Fig. 1b); also, no expression of NAT1 was detected on UV5/NAT2 cells (Fig. 1c).

### Validation of NAT1 and NAT2 specific antibodies

**Figure 2:**
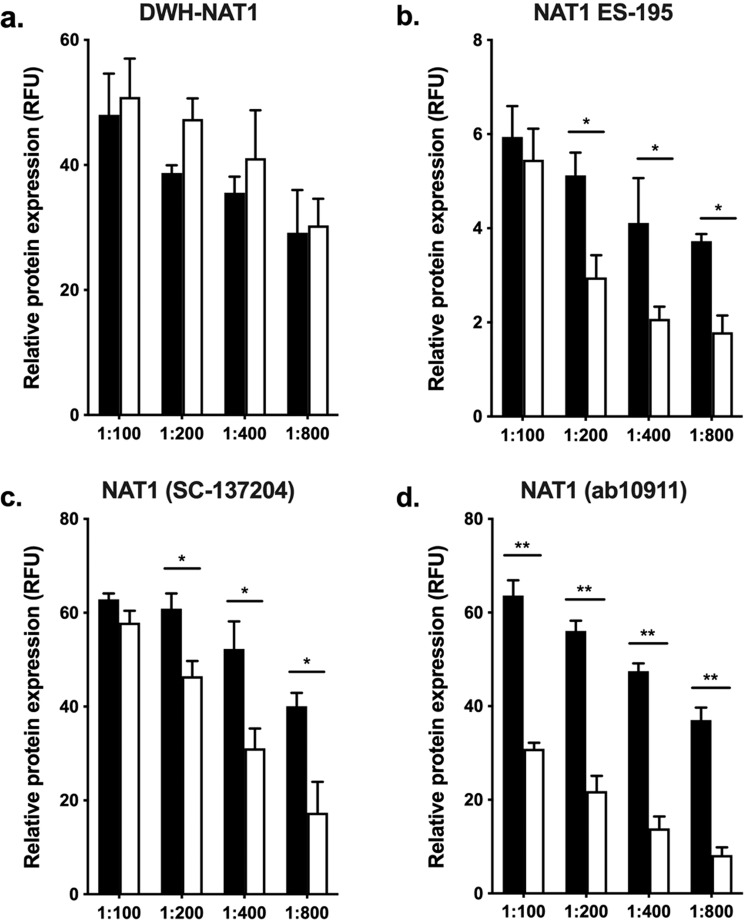
NAT1 protein expression detection in UV5 cells. The relative protein expression of (HUMAN)NAT1 in UV5/NAT1 (black bars) and UV5/NAT2 (clear bars) cells were quantified as described in the methods section. Each bar illustrates the mean ± SEM of relative protein expression from 4 individual determinations. (**a**) NAT1 protein detection using DWH-NAT1 antibody showed no selectivity for NAT protein across all concentrations used (p > 0.05). (**b**) NAT1 protein detection using ES-195 antibody shows specificity for NAT1 in dilutions from 1:200–1:800. (**c**) NAT1 protein quantification using sc-137204 showed specificity NAT1 in a range from 1:200–1:800. (**d**) NAT1 relative expression using ab109114 recombinant monoclonal antibody was 2–4 times more specific for NAT1 across all concentrations tested. *p < 0.05, **p < 0.01.

**Figure 3:**
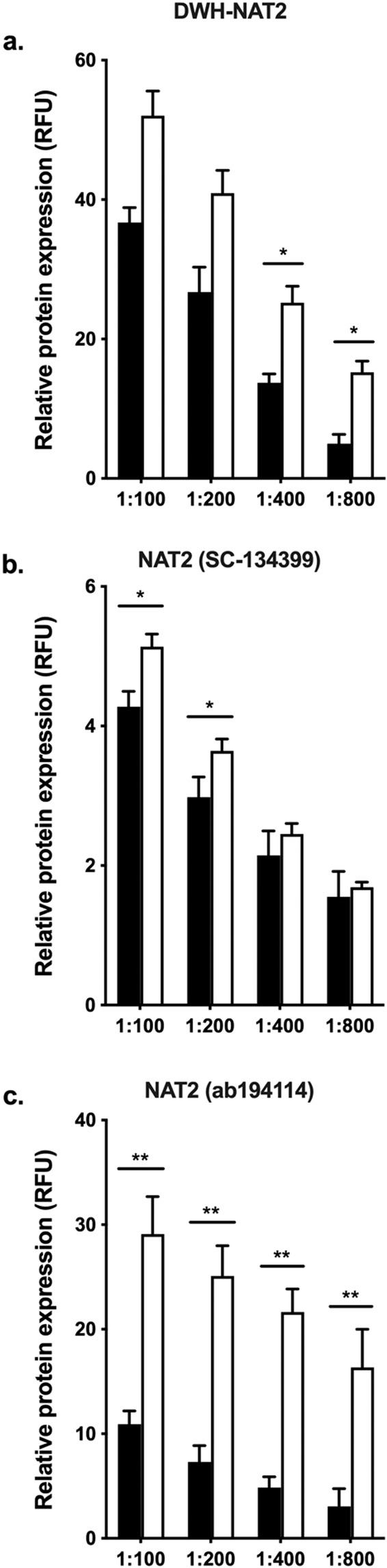
NAT2 protein expression detection in UV5 cells. The relative protein expression of (HUMAN)NAT2 in UV5/NAT1 (black bars) and UV5/NAT2 (clear bars) cells were quantified as described in the methods section. Each bar illustrates the mean ± SEM of relative protein expression from 4 individual determinations. (**a**) Relative NAT2 protein expression using DWH-NAT2 antibody was specific for NAT2 in the range of 1:400–1:800 concentrations, showing 2–3 times more selectivity for NAT2. (**b**) sc-134399 monoclonal antibody showed specificity for NAT2 only at 1:100–1:200 range. (**c**) Expression of NAT2 protein using ab194114 recombinant monoclonal antibody was 3–5 times more selective towards NAT2 across all concentrations. *p < 0.05, **p < 0.01.

Relative NAT1 and NAT2 protein expression were quantified following an in cell western (ICW) protocol, as described in the methods section. The cross reactivity of four NAT1 (Fig. 2) and three NAT2 (Fig. 3) specific antibodies were evaluated in UV5/NAT1 and UV5/NAT2 cell lines. Our data showed no selectivity of DWH-NAT1 antibody (Fig. 2a p > 0.05) for NAT1. Conversely, ES-195 and SC-G5 NAT1 specific antibodies were two times more specific towards NAT1 than NAT2 in dilutions in the range of 1:200 to 1:800 (Fig. 2b,c, p < 0.05); however, the response of SC-G5 is ∼10 times higher than ES-195. Ab109114 recombinant monoclonal antibody showed more selectivity for its target protein in all the dilutions we tested (Fig. 2d); showing ∼2-fold more selectivity at 1:100 and 1:200 dilutions; ∼3-fold at 1:400 dilution and ∼4-fold at 1:800.

The cross-reactivity of the three different NAT2 specific antibodies, showed that DWH-NAT2 was ∼2 times more selective towards NAT2 in a 1:400 dilution: and ∼3 times more selective in 1:800 dilution (Fig. 3a, p < 0.05). SC-2KK1 antibody showed moderate specificity for NAT2 over NAT1 in high concentrations, 1:100 and 1:200 (Fig. 3b, p < 0.05); however, the response of this antibody was significantly lower than other antibodies tested. Finally, ab194114 was more specific for our application, ∼3 to ∼5 times more specific for NAT2 compared to NAT1 in dilutions up to 1:800 (Fig. 3c, p < 0.01).

### SMZ *N*-acetyltransferase activities

**Figure 4:**
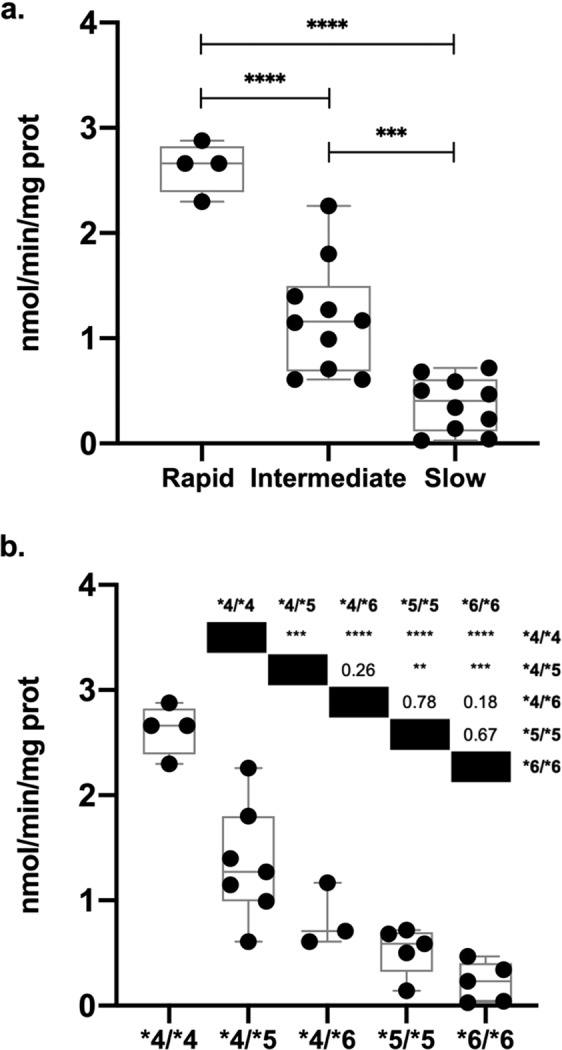
SMZ *N*-acetyltransferase activity in cryopreserved human hepatocytes. SMZ *N*-acetyltransferase activity was quantified as described in the methods section. Each dot represents an individual sample. The line inside the box represents the median *N*-acetyltransferase activity in the corresponding group. (**a**) SMZ *N-*acetyltransferase activity in the rapid, intermediate and slow NAT2 genotypes. (**b**) SMZ *N*-acetyltransferase activity in specific rapid (NAT2*4/*4), intermediate (NAT2*4/*5; NAT2*4/*6) and slow (NAT2*5/*5; NAT2*6/*6) acetylator genotypes. **p < 0.01, ***p < 0.001, ****p < 0.0001.

SMZ *N*-acetyltransferase activity in cryoplateable human hepatocytes was in the relative order rapid> intermediate> slow NAT2 genotype (Fig. 4a). SMZ *N*-acetyltransferase activity differed significantly with respect to NAT2 genotype; rapid vs intermediate (p < 0.0001), intermediate vs slow (p < 0.001), and rapid vs slow (p < 0.0001). When we compared specific NAT2 genotypes (Fig. 4b), NAT2*4/*4 was significantly higher compared to NAT2*4/*5 (p < 0.001), NAT2*4/*6 (p < 0.0001), NAT2*5/*5 (p < 0.0001), NAT2*6/*6 (p < 0.0001). Finally, NAT2*4/*5 hepatocytes were significantly higher than NAT2*5/*5 (p < 0.01) and NAT2*6/*6 (p < 0.001).

### *N-*acetyltransferase 2 mRNA and protein expression in cryopreserved human hepatocytes

**Figure 5:**
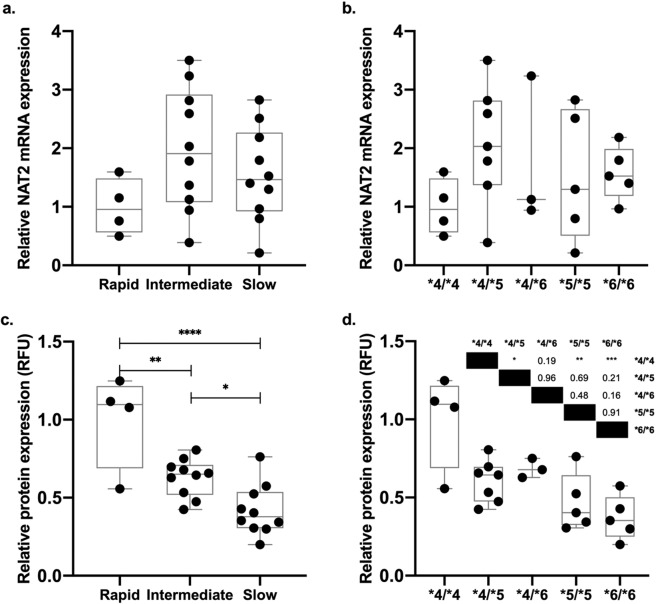
NAT2 mRNA and protein expression in the cryopreserved human hepatocytes. Relative NAT2 mRNA and protein expression were quantified as described in the methods section. Each dot represents an individual sample. The line represents the median relative expression in the corresponding group. (**a**) Relative NAT2 mRNA expression in the rapid, intermediate and slow acetylation genotypes. (**b**) Relative NAT2 mRNA expression in specific rapid (NAT2*4/*4), intermediate (NAT2*4/*5; NAT2*4/*6) and slow (NAT2*5/*5; NAT2*6/*6) acetylator genotypes. (**c**) Relative NAT2 protein expression using monoclonal antibody ab194114, in the cryopreserved human hepatocytes of rapid, intermediate and slow acetylator NAT2 genotypes. (**d**) Relative NAT2 protein expression using monoclonal antibody ab194114, in rapid (NAT2*4/*4), intermediate (NAT2*4/*5; NAT2*4/*6) and slow (NAT2*5/*5; NAT2*6/*6) acetylator genotypes. *p < 0.05, **p < 0.01, ***p < 0.001.

As a part of the present study, we quantified mRNA and protein expression of NAT2 in cryopreserved human hepatocytes as described in the methods section. There was no difference (p > 0.05) in mRNA levels between rapid, intermediate and slow hepatocyte samples (Fig. 5a), or the specific NAT2 genotypes analyzed (Fig. 5b). In contrast, a NAT2 genotype-dependent pattern of protein expression was observed in the human hepatocytes (Fig. 5c, p < 0.0001). NAT2 protein expression was ∼40% and ∼60% lower in the intermediate (p < 0.01) and slow (p < 0.0001) NAT2 genotypes, respectively, vs rapid NAT2 genotypes. We also observed a significant difference between intermediate and slow NAT2 genotypes (p < 0.05). Finally, comparing specific NAT2 genotypes, a significant difference was observed (Fig. 5d, p < 0.001). Rapid acetylator NAT2*4/*4 samples were significantly higher than intermediate acetylator NAT2*4/*5 (p < 0.05) and slow acetylator NAT2*5/*5 (p < 0.01) and NAT2*6/*6 (p < 0.001) NAT2 genotypes.

### Correlation analysis of NAT2 in cryopreserved human hepatocytes

**Figure 6:**
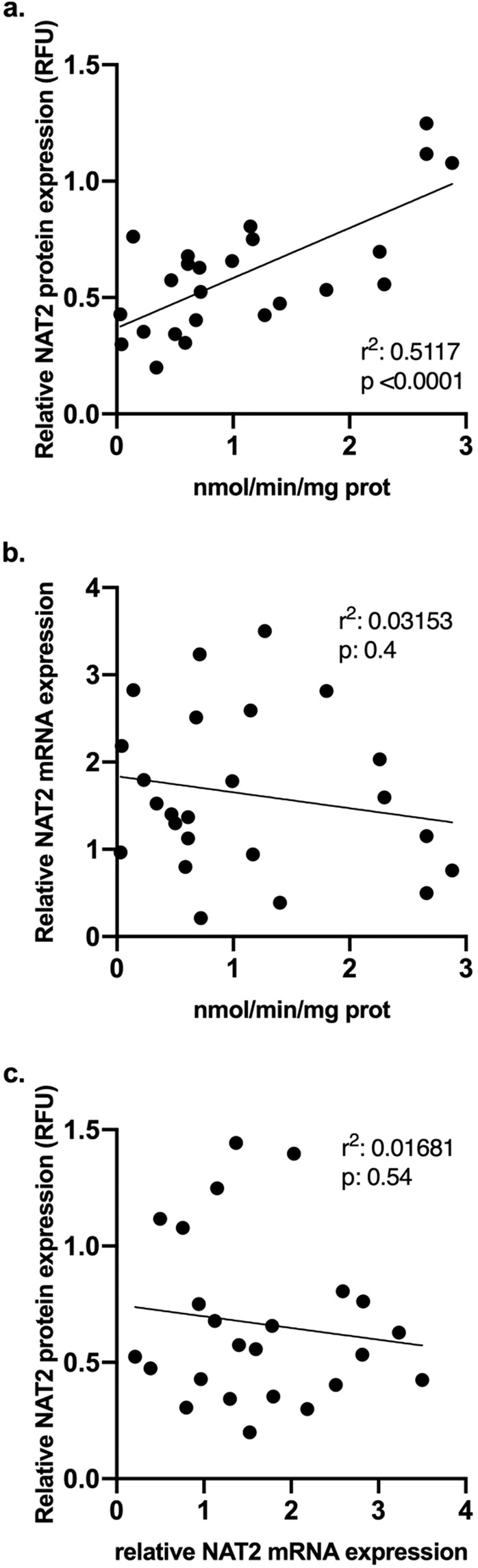
*N*-acetylation activity and protein expression correlation in cryopreserved human hepatocytes. (**a**) Correlation between SMZ *N-*acetyltransferase activity and NAT2 protein expression. (**b**) Correlation between SMZ *N*-acetyltransferase activity and NAT2 mRNA expression. (**c)**. Correlation between NAT2 mRNA expression and NAT2 protein expression.

A moderate correlation was observed between the NAT2 protein expression and SMZ *N*-acetyltransferase activity (Fig. 6a), yielding a determination coefficient of 0.51 (p < 0.0001, 95% CI 0.44–0.87). The correlation coefficients within the rapid, intermediate and slow NAT2 genotypes were 0.65 (95% CI −0.69–0.99), 0.023 (95% CI −0.71–0.53), and 0.0014 (95% CI −0.65–0.61), respectively (Supplementary fig. 1). In contrast, a very slight degree but non-significant correlation was observed between NAT2 mRNA expression and SMZ *N*-acetyltransferase activity (Fig. 6b), and NAT2 mRNA expression vs protein expression (Fig. 6c).

## Discussion

NAT1 and NAT2 are cytosolic proteins that can acetylate a wide variety of arylamine and hydrazine compounds^15^. In the 1960s, Evans and colleagues described differences in response to isoniazid therapy for tuberculosis between a group of patients, this observation led to the description of NAT2 rapid and slow acetylation phenotypes^6^.Rapid, intermediate and slow acetylation phenotypes have been identified^1,16^ based on the presence of specific single nucleotide polymorphisms (SNPs) in NAT2. Individual risks of drug toxicity, therapeutic failure or disease is associated with NAT2 acetylation phenotype^5,17^. SNPs in NAT1 also have been associated with disease risk^1,16^.

NAT1 mRNA and protein expression have been consistently reported to be found across the four different tissues (epithelial, nervous, connective and muscular), as well as, blood cells and major organs^2,4,18^. On the other hand, NAT2 mRNA and protein are primarily expressed in the liver, colon and intestine^3,19^; however, basal levels of expression can be found in blood cells^20^, and during fetal development^21^. Recent reports have discussed the importance of antibody validation for the specific purpose to be used, not doing so, might lead to misinterpretation of the results due to unspecific binding to its target protein^22,23^. Accurate and specific quantification of immunoreactive NAT1 and NAT2 has been challenging based on their high amino acid sequence similarity. The first NAT antibodies were developed after the purification of NAT protein from the chicken liver^24^. Then, the purification of *Salmonella typhimurium* NAT1 crystal structure ((SALTY)NAT1) allowed the generation of more specific antibodies^25^. The purification of (HUMAN)NAT1 allowed the generation of specific antibodies raised against the *C*-terminus portion of the protein, a portion demonstrated to be more immunogenic than the same portion of (HUMAN)NAT2; these antibodies have been used across different studies to report protein expression^26–31^. Our lab has previously developed anti-peptide antibodies against (HUMAN)NAT1 and (HUMAN)NAT2, used for western-blotting assays^32,33^.

In the present work, we compared different antibodies for their ability to quantify immunoreactive NAT1 and NAT2 using In Cell Western method. We investigated the relationship of NAT2 genotype to NAT2 protein expression in cryopreserved human hepatocytes. In order to assess this, we adapted an assay to quantify protein expression in cells fixed to a plate^34,35^. We chose this method because it gives us the ability to quantify the expression of a protein of interest directly in the fixed cells, without the need of protein lysates; thus, simplifying the process compared to the traditional western blotting method. The first step was to find an antibody that provided a good selectivity towards NAT1 or NAT2. The latter was investigated by using UV5 cells expressing either NAT1 or NAT2 and then quantifying the expression of both proteins in the fixed cells. Common western blotting methods rely on SDS-PAGE separation in order to identify immunoreactive proteins based on the specific molecular weight. Since use of the ICW method does not use the separation of proteins based on their molecular weight, the selection of highly specific primary antibodies is crucial to detect a specific protein.

Our data shows that the selectivity of any of the different test antibodies varies depending on the staining conditions used. The (HUMAN)NAT1 ES-195 antibody used in different reports^36,37^, was specific in our assay only if it was diluted at least 200-fold. At higher concentrations, the same antibody was not able to differentiate between NAT1 and NAT2. The same finding is true for other antibodies tested (Fig. 2b,c, respectively). Interestingly, a monoclonal (HUMAN)NAT1 commercial antibody (ab109114) showed high selectivity across all concentrations tested in our assay, making it a viable option for further studies. On the other hand, the anti-peptide (HUMAN)NAT2 antibody generated in our lab (DWH-NAT2) showed high specificity for NAT2 only if it was diluted at least 400-fold. This is consistent with previous reports using the same antibody with a traditional western blotting method^32,38^. Also, a monoclonal (HUMAN)NAT2 commercial antibody (ab194114) was significantly selective against NAT2 in all concentrations tested (Fig. 3a,c, respectively). Our data replicate the results obtained in previous reports using a traditional western blotting method^32,36–38^. Since NAT1 is an exceedingly high specific activity enzyme that is present at extremely low protein levels in extracts from human tissues and primary cells (beneath the level of detection of any Western method unless a substantial pre-purification step is incorporated), NAT1 in the primary hepatocytes may be undetectable and therefore non-interfering, regardless of the selectivity of the NAT antibody used. The data we obtained for different commercially available antibodies are valuable for the development and interpretation of future studies.

A trimodal distribution of NAT2 *N*-acetylation capacity in human populations has been shown for different compounds like sulfamethazine (SMZ)^39,40^, isoniazid (INH)^41,42^ and sulfasalazine^43^. Also, in animal models such as congenic mouse^44^, Syrian hamster^45,46^, and rat^47,48^. We used cryoplateable human hepatocytes as a model, it showed a clear separation in the *N*-acetylation capacity of the three different phenotypes, very similar to previous reports using cryoplateable human hepatocytes using SMZ as substrate^33^. In a previous report by our group, genetic heterogeneity among slow acetylator cryopreserved human hepatocytes was observed^49^; in the present study we observed a similar trend, even though no statistical significance was observed (p > 0.05).

Our data shows also a clear NAT2 protein expression difference between rapid, intermediate and slow acetylator human hepatocytes; being higher in the rapid, compared to the intermediate and the slow (ANOVA p < 0.0001). When we compared the expression in specific NAT2 genotypes, NAT2*4/*4 was significantly higher than NAT2*5/*5 or NAT2*6/*6 (p < 0.01, p < 0.001, respectively). A reduction in the NAT2 protein expressed in human liver from individuals with slow acetylator phenotype has been reported previously^14,19,41^. Some NAT2 alleles (including NAT2*5B and NAT2*6 A) recombinantly expressed in COS-1 cells showed reduced levels of immunoreactive NAT2 protein compared with NAT2*4^19,32^. Previous reports have demonstrated the effect of different SNPs in the protein stability or susceptibility to degradation. For example rs1801280 (341 T > C) present in NAT2*5 alleles, causes Ile^114^Thr, the substitution of a non-polar (Ile) for a polar (Thr) amino acid residue that results in a hydrophobicity change that in turn causes the protein more susceptible for degradation, and thus less detection of immunoreactive protein is observed^50^. On the other hand, SNP rs1799930 (590 G > A), present in NAT2*6 alleles, results in a substitution of Arg^197^Gln. Based on functional studies^32,51^, this modifies the charges of the side chain of the corresponding domain, thus affecting the catalytic activity and protein level due to a reduction of protein thermostability. For both of these SNPS, the effect of protein level reduction is due to structural changes in the protein, not to mRNA regulation which is consistent with our findings in cryopreserved human hepatocytes. Finally, our data show a good correlation between NAT2 protein expression and *N*-acetylation activity in cryopreserved human hepatocytes.

In conclusion, we compared methods for the quantification of immunoreactive NAT1 and NAT2 with seven different antibodies and investigated the relationship of NAT2 genotype to NAT2 mRNA and protein expression in cryopreserved human hepatocytes. We describe a specific analytical method for the quantification of NAT1 and NAT2 protein expression and showed that NAT2 activity in human hepatocytes was directly correlated to the expression levels of NAT2 protein but not mRNA.

## Materials and methods

### University approval for experimental protocols

All experimental protocols described below were carried out in accordance with the relevant guidelines and approval of the University of Louisville Institutional Biosafety Committee.

### Cell culture

The construction of UV5-CHO cells expressing NAT1*4 (reference NAT1 allele) or NAT2*4 (reference NAT2 allele) is described elsewhere^52,53^. Briefly, UV5‐CHO cells, a nuclease excision repair (NER)‐deficient derivative of AA8 which are hypersensitive to bulky DNA lesions, were obtained from the ATCC (catalogue number: CRL‐1865). Cells were incubated at 37 °C in 5% CO_2_ in complete alpha‐modified minimal essential medium (α‐MEM, Walkersville, MD) without L‐glutamine, ribosides, and deoxyribosides supplemented with 10% fetal bovine serum (Hyclone, Chicago, IL, USA), 100 units/mL penicillin, 100 µg/mL streptomycin, and 2 mM L‐glutamine (Walkersville, MD ). The UV5/CHO cells used in this study were previously stably transfected with a single Flp recombination target (FRT) integration site^52^. The FRT site allowed stable transfections to utilize the Flp‐In System (Invitrogen, Carlsbad, CA, USA). When co-transfected with pOG44 (Invitrogen, Carlsbad, CA, USA), a Flp recombinase expression plasmid, a site‐specific, conserved recombination event of pcDNA5/FRT (containing either NAT1*4 or NAT2*4) occurs at the FRT site. The FRT site allows recombination to occur immediately downstream of the hygromycin resistance gene, allowing for hygromycin selectivity only after Flp‐recombinase mediated integration. They are referred to in this manuscript as UV5/NAT1 or UV5/NAT2 cells.

### Source and culture of cryoplateable human hepatocytes

Cryoplateable human hepatocyte samples obtained from a commercial vendor (BioIVT; Baltimore, MD, USA) who collected the samples from donors under IRB approved protocols (http://www.bioivt.com/) to be used for experimental research. Hepatocytes were prepared from fresh human tissue; isolated and frozen within 24 h of organ removal. All hepatocyte samples are human transplant rejected and tested negative for hepatitis B and C and HIV 1 and 2. We followed the cell culture and lysis protocol reported in^33^. Briefly, upon removal from liquid nitrogen, hepatocytes were thawed according to the manufacturer’s instructions and the cell suspension centrifuged for 5 min. The supernatant was discarded, and cells were washed before lysing the cells. The lysate was centrifuged at 15,000 ×g for 20 min and the supernatant was aliquoted and stored at − 80 °C. Protein concentrations in the lysates were determined using the Bio-Rad protein assay kit (Bio-Rad, Hercules, CA, USA).

### NAT2 genotyping and assignment of acetylator phenotype

Genomic DNA was isolated from pelleted cells prepared from human cryoplateable hepatocyte samples as described previously by using the QIAamp DNA Mini Kit (QIAGEN, Valencia, CA, USA) according to the manufacturer’s instructions. NAT2 genotypes and deduced phenotypes were determined as previously described^38^. Individuals possessing two NAT2 alleles associated with rapid acetylation activity (NAT2*4, NAT2*12, and NAT2*13) were classified as rapid acetylators; individuals possessing one of these alleles and one allele associated with slow acetylation activity (NAT2*5, NAT2*6, NAT2*7, and NAT2*14) were classified as intermediate acetylators, and those individuals that possessed two slow acetylation alleles were classified as slow acetylators. For the present study, 24 samples were randomly selected; 5 different haplotypes were selected, NAT2*4/*4 (4 samples), NAT2*5/*5 (5 samples), NAT2*6/*6 (5 samples), NAT2*4/*5 (7 samples) and NAT2*4/*6 (3 samples) that totals 4 rapid, 10 intermediate and 10 slow acetylation phenotypes.

### NAT1 and NAT2 mRNA expression

Total RNA was isolated from cells using the E.Z.N.A. Total RNA Kit I (Omega Bio-Tek, Norcross, GA, USA) followed by removal of contaminating DNA by treatment with TURBO DNA-*free* Kit (Thermo Fisher Scientific, Waltham, MA, USA). Synthesis of cDNA was performed with High-Capacity cDNA Reverse Transcription Kit (Thermo Fisher Scientific, Waltham, MA, USA) using 1 μg of total RNA in a 20 μL reaction per the manufacturer recommendations. Quantitative RT-PCR (RT-qPCR) assays were used to assess the relative amount of NAT1 or NAT2 mRNA in cells in the UV5/NAT1 and UV5/NAT2 cells, as well as cryoplateable hepatocytes. The Step One Plus (Thermo Fisher Scientific, Waltham, MA, USA) was used to perform RT-qPCR in reactions containing 1× final concentration of iTaq Universal SYBR Green Supermix (Bio-Rad, Hercules, CA, USA), 500 nM of each primer (FW: 5′-GAATTCAAGCCAGGAAGAAGCA-3′, RV: -TCCAAGTCCAATTTGTTCCTAGACT-3′), in a total volume of 20 μL. An initial incubation at 50 °C was carried out for 2 min and at 94 °C for 10 min followed by 40 cycles of 95 °C for 15 s and 60 °C for 1 min. β2-Microglobulin (B2M, FW: 5′-AGTCAACTTCAATGTCGGATGGAT-3′, RV: 5′-CCTGGAGGCTATCCAGCGTAC-3′), was used to determine ΔCt (NAT1 or NAT2 Ct - B2M Ct). ΔΔCt was determined by subtraction of the smallest ΔCt and relative amounts of NAT1 mRNA were calculated using 2^−ΔΔCt^ as previously described^18^.

### NAT1 and NAT2 Antibodies

Seven different antibodies were investigated for their specificity for NAT1 or NAT2. Two antibodies, anti-NAT1 rabbit polyclonal and anti-NAT2 rabbit polyclonal were custom designed and obtained from BioSource International (now part of Thermo Fisher Scientific, Waltham, MA, USA), referred to as DWH-NAT1 and DWH-NAT2, respectively. DWH-NAT1 immunogen sequence is CLHSDLLEDSKYR. DWH-NAT2 immunogen sequence is FLNSHLLPKKKHQ^50,54^. Briefly, the corresponding sequences for each of these antibodies were conjugated to KLH prior immunization. Then, following 1 immunization plus 4 boosts in 2 different animals; the serum was obtained and then purified using affinity columns. Other antibodies investigated were NAT1 rabbit polyclonal antiserum ES-195 (kindly provided by Professor Edith Sim, University of Oxford, UK), NAT1/2-G5 mouse monoclonal sc-137204 and NAT2 (4-KK21) mouse monoclonal sc-134399 (Santa Cruz Biotechnology, Dallas, TX, USA), recombinant anti-NAT1 rabbit monoclonal antibody [EPR3221(2)] (ab109114), and recombinant anti-NAT2/AT-2 rabbit monoclonal antibody [EPR15856] (ab194114) (Abcam, Cambridge, UK).

### NAT1 and NAT2 in-cell western staining in UV5/NAT1 and UV5/NAT2 cells

UV5/NAT1, UV5/NAT2 cells (1×10^5^) were plated into 96-well black/clear bottom plates (Thermo Fisher Scientific, Waltham, MA, USA) and incubated overnight at 37 °C and 5% CO_2_. A modification of the staining protocol reported in^35^, was used. Briefly, once attached to the plate, cells were fixed to the plate with 3.7% formaldehyde in TBS solution. After fixing, cells were permeabilized using 0.1% Triton-X-100 in TBS solution for 5 minutes, this process was repeated four times. The blocking of the cells was done using Fish Gelatin Agent in TBS (Biotium, Fremont, CA, USA) for 1.5 hours. After blocking, cells were incubated with different NAT1 or NAT2 antibodies previously described in decreasing dilution ratios from 1:100 to 1:800, and β-actin (A2228 (1:200)), Sigma-Aldrich, St. Louis, MO, USA) overnight at 4 °C with constant agitation. After primary antibodies incubation, plates were washed five times with 0.1% Tween 20 in TBS for 5 min. Secondary detection was carried out using IRDye 800CW Goat anti-Rabbit IgG (1:1200), IRDye 800CW Goat anti-Mouse IgG (1:1200) or IRDye 680RD Goat anti-Mouse IgG (1:1200), (LI-COR Biosciences, Lincoln, NE, USA) incubation for 60 min. Finally, cells were washed with Tween solution as described previously. NAT1 or NAT2 and β-actin were simultaneously visualized using an Odyssey infrared imaging Scanner (LI-COR Biosciences, Lincoln, NE, USA) using the 680 nm channel and 800 nm channel. Relative fluorescence units (RFU) allowed a quantitative analysis. Relative protein expression was calculated by dividing RFU for NAT1 or NAT2 (800 nm channel) by the RFU of β-actin (680 nm channel).

### NAT2 in-cell western staining in cryoplateable human hepatocytes

Cryoplateable human hepatocytes (2×10^5^ viable cells) were plated into 96-well black/clear bottom plates (Thermo Fisher Scientific, Waltham, MA, USA) and incubated overnight at 37 °C and 5% CO_2_ on InVitroGRO HT medium (BioIVT, Baltimore, MD, USA). Once attached to the plate, cells were fixed, permeabilized and blocked following the same protocol previously described for UV5 cells. After blocking, cells were incubated with NAT2 specific antibody (ab194114 (1:200)), and β-actin (A2228 (1:200)), Sigma-Aldrich, St. Louis, MO, USA) overnight at 4 °C with constant agitation. Following primary antibodies incubation, the same protocol previously described for UV5 cells was followed for secondary antibodies incubation and detection.

### **Measurement of*N-*acetyltransferase 2 activity**

SMZ *N*-acetyltransferase activity was measured using modifications of high-performance liquid chromatography (HPLC) assays as previously described^55,56^. Suitably diluted hepatocyte lysate, acetyl coenzyme A (1000 µM) and SMZ (300 µM) were incubated at 37 °C for 10 min. The reaction was stopped by the addition of 1 M perchloric acid. Following centrifugation to precipitate protein, reaction supernatants were injected onto an Infinity 1260 system (Agilent Technologies, Santa Clara, CA) using an EM Science 125 mm × 4 mm Lichrocart C18 (5 µm) column fitted with a similar Lichrocart guard column (4 mm × 4 mm) (Merck, Darmstadt, GER). Reactants and products were eluted from the column with a 10-min linear gradient (2 ml/min) from 100% sodium perchlorate (pH 2.5) to 100% acetonitrile. Protein concentrations in the lysates were determined using the Bio-Rad protein assay kit (Bio-Rad, Hercules, CA, USA). *N*-acetyltransferase activities were calculated as nanomoles of *N*-acetyl-SMZ product per minute per mg of cytosolic protein.

### Statistical analysis

Statistical differences in NAT1 (Fig. 2) or NAT2 (Figs. 3–5) expression were determined using an unpaired student’s t-test or one-way ANOVA followed by Tukey post-hoc test as appropriate. All statistical analyses were performed using GraphPad Prism v8.2.1 (GraphPad Software, San Diego, CA, USA). The results are expressed as the mean ± the standard error of the mean (SEM). Values of p < 0.05 were considered statistically significant.

## Supplementary information

Supplementary Information

## Data Availability

The datasets generated during and/or analyzed during the current study are available from the corresponding author on reasonable request.
