# Supplementary material for: Human arylamine N-acetyltransferase 2 genotype-dependent protein expression in cryopreserved human hepatocytes
Source: Sci Rep. 2020 May 5;10:7566. doi: 10.1038/s41598-020-64508-0 (PMC7200704; doi:10.1038/s41598-020-64508-0)
Supplement: Supplementary file 1 — Supplementary Information [file 41598_2020_64508_MOESM1_ESM.docx]

**Human arylamine *N*-acetyltransferase 2 genotype-dependent protein expression in cryopreserved human hepatocytes**

Raúl A. Salazar-González, Mark A. Doll and David W. Hein*

Department of Pharmacology & Toxicology and James Graham Brown Cancer Center,

University of Louisville School of Medicine, Louisville, Kentucky 40202 USA

Corresponding Author

*David W. Hein, Department of Pharmacology and Toxicology, Kosair Charities CTR-Room 303, 505 South Hancock Street, Louisville, KY, USA, 40202, david.hein@louisville.edu.


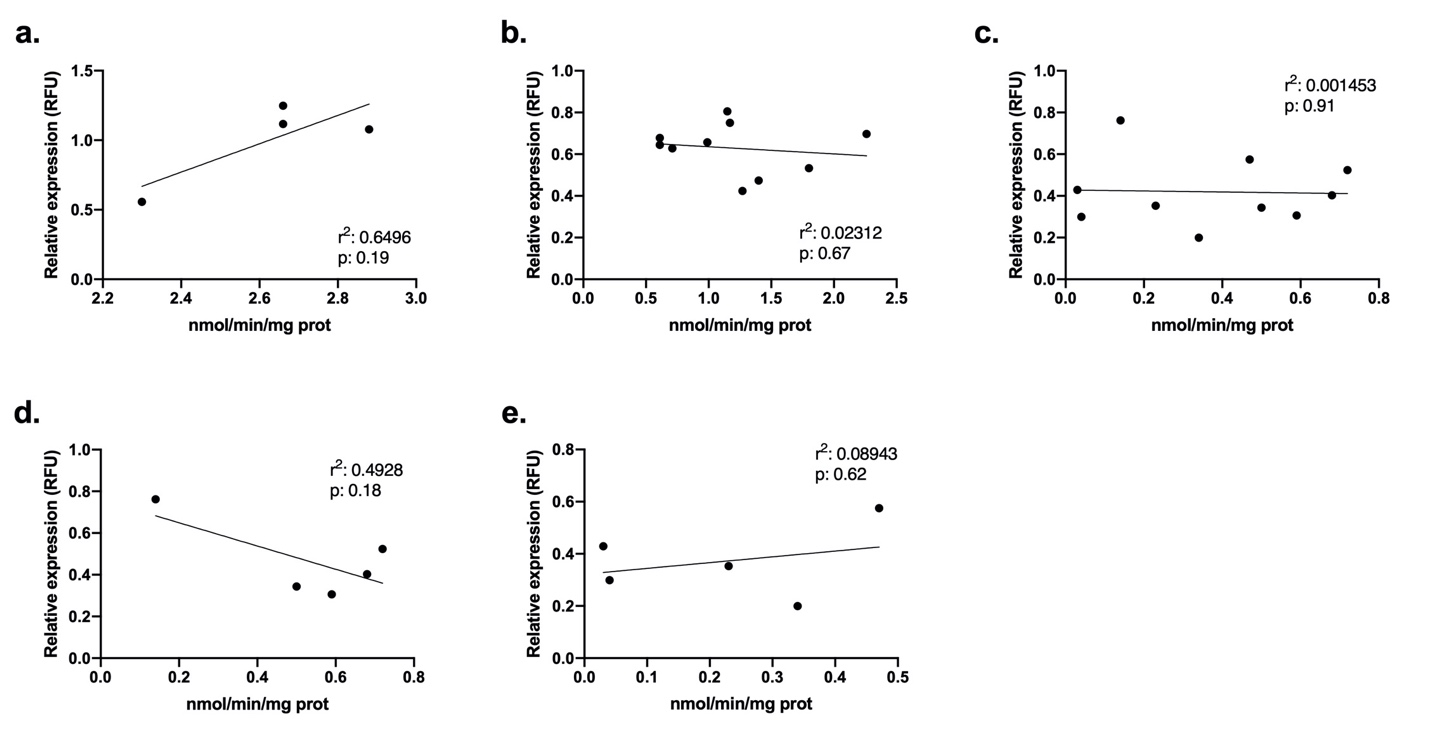


**Supplemental Figure 1.** **Correlation analysis of *N*-acetylation activity and protein expression in cryopreserved human hepatocytes based on their NAT2 genotype or inferred phenotype**. **a.** Rapid (*NAT2*4/*4*) acetylators. **b.** Intermediate (*NAT2*4/*5, NAT2*4/*6*) acetylators. **c.** Slow (*NAT2*5/*5, NAT2*6/*6*) acetylators. **d.** *NAT2*5/*5* acetylators. **e.** *NAT2*6/*6* acetylators.
